# A systematic review of mental health outcome measures for young people aged 12 to 25 years

**DOI:** 10.1186/s12888-015-0664-x

**Published:** 2015-11-14

**Authors:** Benjamin Kwan, Debra J. Rickwood

**Affiliations:** 1 Faculty of Health, University of Canberra, Kirinari Street, Bruce, ACT 2601 Australia; 2 Headspace National Youth Mental Health Foundation National Office, 485 La Trobe Street, Melbourne, VIC 3000 Australia

**Keywords:** Youth, Young people, Mental health, Change, Routine outcome measure, Feedback

## Abstract

**Background:**

Mental health outcome measures are used to monitor the quality and effectiveness of mental health services. There is also a growing expectation for implementation of routine measurement and measures being used by clinicians as a feedback monitoring system to improve client outcomes. The recent focus in Australia and elsewhere targeting mental health services to young people aged 12–25 years has meant that outcome measures relevant to this age range are now needed. This is a shift from the traditional divide of child and adolescent services versus adult services with a transitioning age at 18 years. This systematic review is the first to examine mental health outcome measures that are appropriate for the 12 to 25 year age range.

**Methods:**

MEDLINE and PsychINFO databases were systematically searched to identify studies using mental health outcome measures with young people aged 12 to 25 years. The search strategy complied with the relevant sections of the PRISMA statement.

**Results:**

A total of 184 published articles were identified, covering 29 different outcome measures. The measures were organised into domains that consisted of eight measures of cognition and emotion, nine functioning measures, six quality of life measures, and six multidimensional mental health measures. No measures were designed specifically for young people aged 12 to 25 years and only two had been used by clinicians as a feedback monitoring system. Five measures had been used across the whole 12 to 25 year age range, in a range of mental health settings and were deemed most appropriate for this age group.

**Conclusions:**

With changes to mental health service systems that increasingly focus on early intervention in adolescence and young adulthood, there is a need for outcome measures designed specifically for those aged 12 to 25 years. In particular, multidimensional measures that are clinically meaningful need to be developed to ensure quality and effectiveness in youth mental health. Additionally, outcome measures can be clinically useful when designed to be used within routine feedback monitoring systems.

**Electronic supplementary material:**

The online version of this article (doi:10.1186/s12888-015-0664-x) contains supplementary material, which is available to authorized users.

## Background

An outcome measure in mental health care can be defined as a tool used to measure the effect on a person’s mental health as a result of health care intervention, plus any additional extra-therapeutic influences [1]. Specifically, outcome measures are quantitative indicators used at two or more points in time: baseline, post-intervention, discharge, or follow-ups [2, 3]. Routine outcome measurement, whereby the same outcome measure is used frequently at a number of time points, has been adopted in child and adolescent mental health services in Australia, New Zealand, Denmark, United Kingdom and Norway [4]. This push has been driven by an increasing emphasis on monitoring the quality and effectiveness of services [5, 6].

Routine outcome measurement reported at the service level enables decision making around funding of services, particularly at a government level where health resources are limited and need to be distributed to achieve the best outcomes [4]. It is also essential as a component of ongoing service-level quality improvement. Importantly, routine outcome measurement improves clinical practice when it is part of a feedback monitoring system for clinicians [7]. When mental health measures are regularly provided to the clinician they can inform clinical decision making and enable the clinician to adjust treatment planning accordingly [8]. In adult mental health services feedback has been shown to increase accuracy of diagnosis, improve communication between client and clinician, enhance treatment monitoring, and help clients maintain positive effects for longer periods [9, 10]. For clients who are not improving or who are deteriorating during therapy, feedback systems can help improve outcomes [9]. Emerging research in youth mental health contexts suggests similar benefits of feedback monitoring systems for younger clients [11, 12].

To be useful, mental health outcome measures must be valid and reliable, sensitive to change, comparable across relevant client groups and service types, and meaningful to both clients and clinicians [6]. Fundamentally, outcome measures must be sensitive to change and be able to clearly convey the magnitude of change achieved [13]. However, measuring change is complex and needs to go beyond reporting statistical significance. Effect sizes and the timescale in which the change is evident are essential [14]. An increasingly used technique is calculating a measure of reliable change, which takes into account the reliability of the measurement instrument and has been proposed to provide a more accurate standard of meaningful change [15, 16]. Additionally, estimating clinical significance, which is distinct from statistical significance, has been recommended in mental health contexts. Change is clinically significant when a client moves from the dysfunctional to the functional range during therapy. This technique is not commonly used as it requires comparison populations and norms [17]. These metric are useful, however, as a client can be considered “recovered” when their outcomes show both reliable change and clinical significance [16]. Meaningful changes are also those that are of value and considered important by the client, family or clinician [18, 19]. Notably, quantifiable change can be different from perceived change, which means that it is important to determine outcome measures that are personally meaningful to clients [20].

Outcome measures need to be comparable over relevant client groups and treatment settings, and help inform initial case formulation and client prioritisation access. Outcome measures are increasingly designed to measure broad mental health status rather than assess symptoms associated with the diagnosis of specific mental disorders [21]. Specific measures may be required for diagnosis, but are not helpful when making comparisons between cases and services where differences in case mix exist [22]. Using specific diagnostic measures also means clinicians need to isolate a particular presenting problem at baseline to assess subsequent change. This presents challenges for the common situations when clients have comorbid mental health issues or their presenting issues change over the course of therapy [11, 23]. In contrast, measures of general mental health can be used in a range of mental health settings with different client characteristics, including public mental health agencies, private organisations, schools, and hospitals. Being generically relevant to a broad range of mental health presentations enables the measure to cater for clients with no disorder, such as those accessing prevention mental health programs, through to those with severe disorder, such as inpatient hospital clients [24]. It is important to note the role of outcome measures in epidemiological studies to track naturalistic change in non-clinical populations.

To be clinically useful, outcome measures need to be meaningful to clients and relevant to the areas in which they have treatment goals. Research with mental health service consumers shows that many measures are not particularly relevant to their situations and do not capture outcomes that are personally meaningful [25]. Determining an outcome measure that is applicable in both clinical work and service evaluation is challenging [26]. Mental health is a broad construct that comprises a number of different measurement domains [27]. These include measures that cover recovery, cognitive performance and emotional experience, ability to undertake daily activities and maintain interpersonal relationships consistent with development stage, and general life satisfaction and wellbeing [1, 28, 29]. Each domain has been recognised as providing a meaningful aspect of a client’s mental health status, but may vary in value for clinical use, service evaluation and epidemiological studies [27].

There is a long history of outcome measures for adult mental health services and for child and adolescent services, including both community-based and inpatient settings. In Australia, a comprehensive report on outcome measurement in community settings identified 136 measures, of which 31 were deemed most appropriate and being relevant for children and adolescents, adults or older persons [29]. The measures incorporate both client and clinician reporters, and parent reporter measures were available for children and adolescents [22]. Historically, outcome measures have either been targeted towards children and adolescents or adults, reflecting the traditional demarcations within the mental health care system [30]. For example, the Health of the Nation Outcome Scales (HoNOS) has two versions, one for adults aged 18 to 64 years and a child and adolescent version (HoNOSCA) for those aged under 18 years [31]. Outcome measures specific to the youth transition period of adolescence and young adulthood are urgently needed due to recent changes in mental health service delivery specifically targeting this age range [32].

Reorienting mental health services to focus on young people is supported by understanding that they have the highest burden of mental illness across the lifespan, comprising 55 % of the burden of illness for the 15 to 24 year old age group [33]. At least one in four young people aged 12 to 24 years experiences a mental health problem in any given year [34]. Research indicates that 75 % of people suffering from a psychiatric disorder in adulthood experience onset by the age of 24 [35]. Of particular concern, however, young people are least likely to access support from mental health care organisations [32]. A systematic review of barriers and facilitators to mental health help-seeking in young people from qualitative studies identified the major barriers as problems recognising symptoms, a preference for self-reliance, and perceived stigma and embarrassment [36]. There is also a pervasive belief among young people that seeking help does not help [37]. Consequently, ways to ensure mental health support is effective, and perceived to be so, are essential to engage young people in services [38], and this requires being able to demonstrate meaningful outcomes from young people’s mental health service use [39].

Due to increased vulnerability to mental disorder during adolescence and early adulthood, the transition from child and adolescent to adult mental health services at the age of 18 years is extremely disruptive to effective mental health care; it undermines continuity of care at the time when this needs to be strongest [40]. Early intervention youth mental health initiatives are strongly promoted in Australia [41] and gaining momentum in many other countries [42]. Youth-focused service innovations focus on the importance of factors such as youth participation, shared decision making, and easy early access. This has led to the development of tools and supports aimed at engaging young people, such as age appropriate psychosocial and mental health assessments [43]. New methods of delivering mental health interventions to young people have also emerged, which include online and smart phone applications of counselling, self-help, assessment, and support groups [44].

Consequently, appropriate outcome measures are now required that are appropriate to young people’s developmental, social and emotional stages [45, 46]. The current study comprised a systematic review to identify appropriate mental health outcome measures for young people aged 12 to 25 years. Specifically, the review aimed to identify outcome measures that could be used for a broad range of mental health presentations and assessed mental health through global measures of cognition and emotion, functioning, quality of life and multidimensional factors (rather than focussed on specific diagnostic symptoms). The review aimed to explore how outcome measures have been used to track change, in what populations and settings they have been used, and whether they have been used as a feedback monitoring system to clinicians.

## Methods

### Search strategy

The search was conducted using the MEDLINE and PsychINFO databases, covering studies published since the inception of each database until the 9^th^ June 2014. The search terms comprised four categories: young people, measures, mental health and change (see Table 1). These were combined using ‘and’ statements and searches were performed on article titles, abstracts and subjects. Additional studies were identified through hand searching the references of relevant studies and reviews. The search methodology and reported findings comply with the relevant sections of the Preferred Reporting Items for Systematic Reviews and Meta-Analyses (PRISMA) statement [47]. See Additional file 1 for PRISMA checklist.

**Table 1:** Search terms

Categories	Words & phrases
Young person	Young, youth*, adolescen*
Measures	Measure, assessment, rating, scale, screen, questionnaire, checklist, tool
Mental health	Mental health, mental illness, mental disorder, emotional problems, top problems, psychological adjustment, psychological distress, psychiatric disorder, well-being, global functioning, quality of life
Change	Change, improve*, progress

*Is a wildcard character that may be used in place of any number of characters in a search word

### Eligibility criteria

The eligibility criteria included articles reporting global measures of mental health, used with a range of mental health populations for young people aged 12 to 25 years, and measuring change over time. Case studies, reviews, single study specific outcome measures and studies including participants with other medical conditions were excluded. To be included, studies had to:

- be written in English;
- include participants with a mean age in the range of 12 to 25 years;
- describe an outcome measure used as a general measure of mental health, including measures of emotion and cognition, functioning, quality of life and multidimensional mental health;
- report outcome measures tracking change over at least two measurement time points; and
- be applicable to a general mental health population or used with a variety of specific mental health populations (rather than be unique to a particular mental disorder or condition).

Additionally, the criteria excluded studies:

- of only adult or child participants;
- that were case studies or reviews;
- where participants had conditions related to physical health, developmental delays, neurological impairments, intellectual disabilities, learning disorders, situational stress/trauma and substance or alcohol dependence; and
- which had an outcome measure that was single study specific.

### Data extraction

**Fig. 1:**
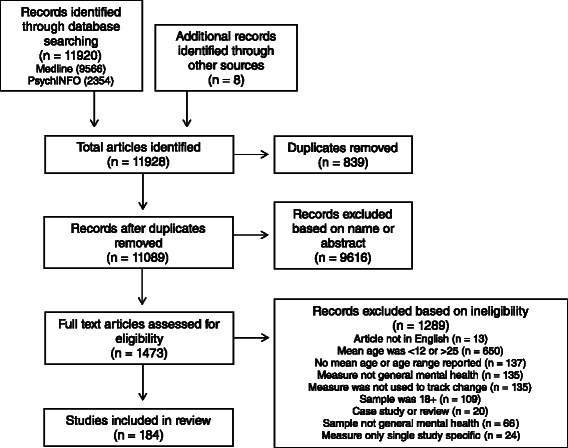
PRISMA flow diagram

Following the database search, duplicates were firstly removed. Titles and abstracts were then screened and irrelevant studies removed. Full text articles of studies identified as possibly relevant for inclusion were then obtained and both authors inspected these against the eligibility criteria for inclusion. The database search was extensive, but authors of the published articles were not contacted to obtain further information to that published. Additional searching by name of each outcome measure identified in the review was not conducted as the aim was to identify outcome measures that met the eligibility criteria rather than identify every published article on the identified measures. Figure 1 shows the PRISMA flow diagram for study inclusion.

Relevant information from each article was entered into a spreadsheet that included: age, gender, ethnicity, socioeconomic status, country, diagnosis, sample size, research design, setting, time of follow-up, reporter, measure change magnitude, and use in feedback monitoring systems. The articles were then sorted into groups by the outcome measure(s) identified in the article. If more than one eligible measure was reported, the article was included under each relevant outcome measure group. Lastly, the outcome measures were categorised according to the major domains of cognition and emotion, functioning, quality of life or multidimensional.

## Results

### Search results

The search strategy identified 184 published articles covering 29 different outcome measures, with many articles identifying more than one measure. The key characteristics of each article by type of measure are summarised in Additional file 2. The outcomes comprised eight measures of cognition and emotion, nine of functioning, six that were quality of life, and six multidimensional mental health measures. The GAF, a measure of functioning, was the most commonly referenced measure overall. The most referenced measure of cognition and emotion was the CBCL; for quality of life, it was the SF-36; and the most referenced multidimensional measure was the HoNOSCA.

### Age range

**Fig. 2:**
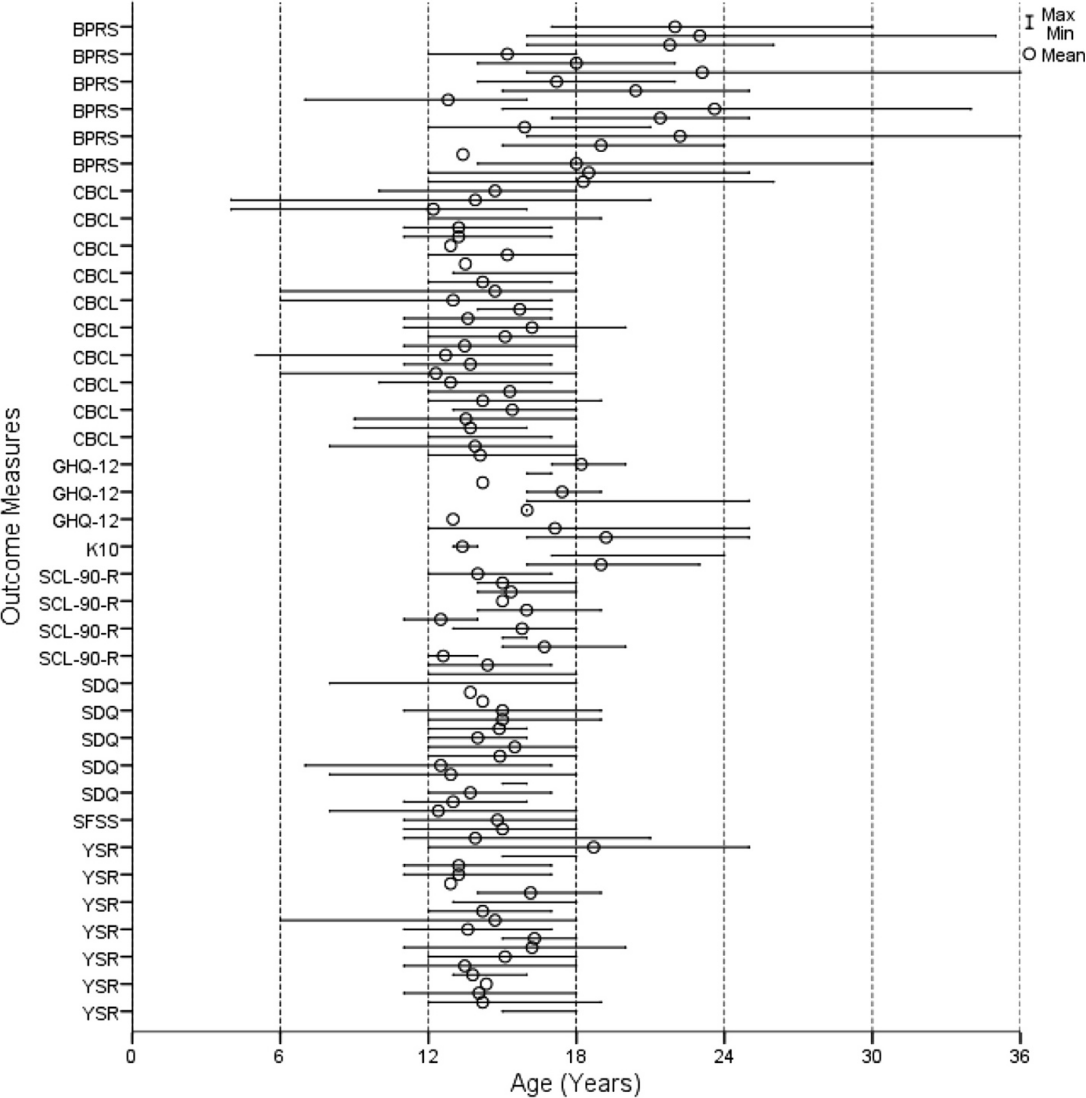
Age range and mean of cognition and emotion measures

**Fig. 3:**
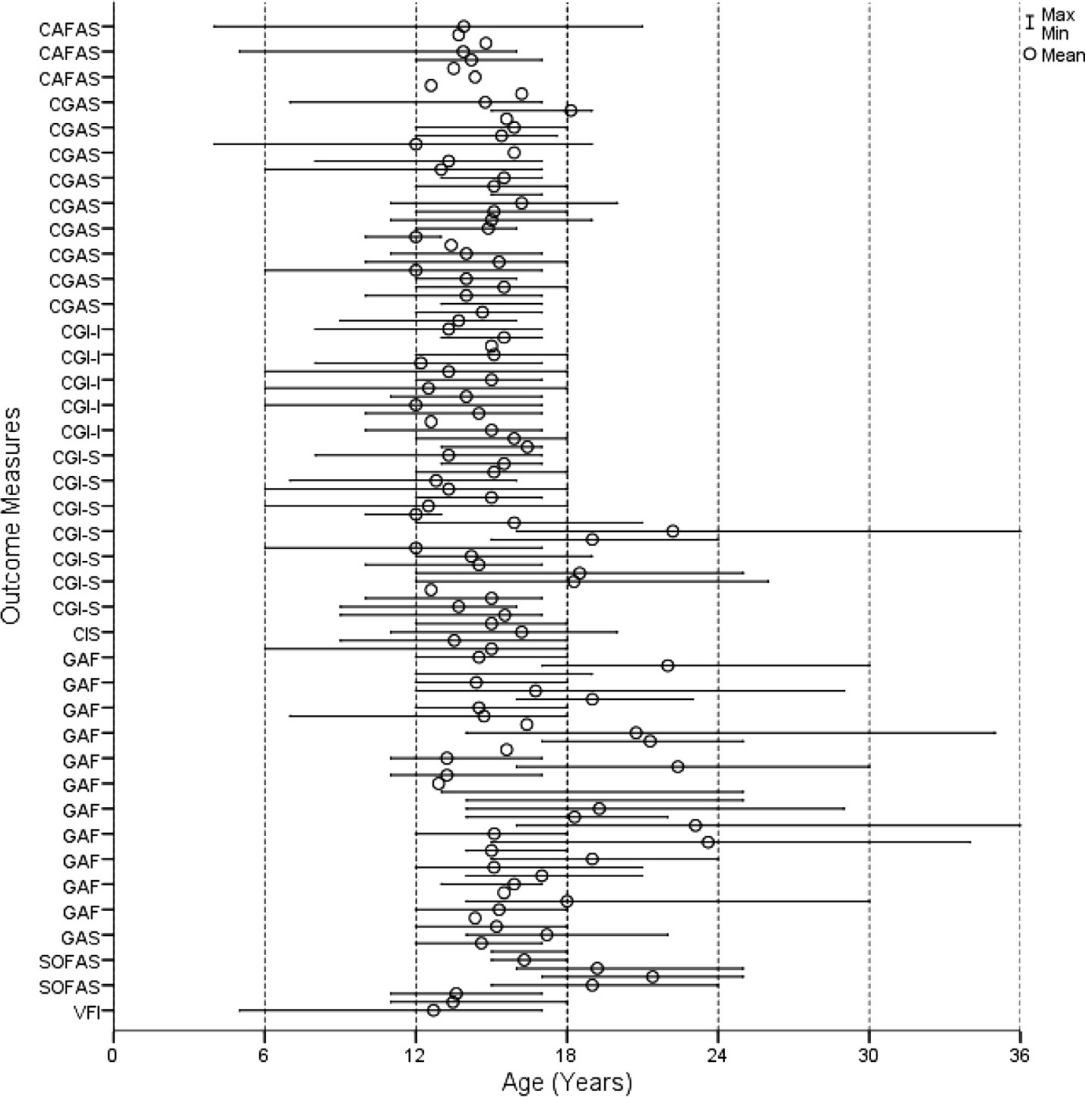
Age range and mean of functioning measures

**Fig. 4:**
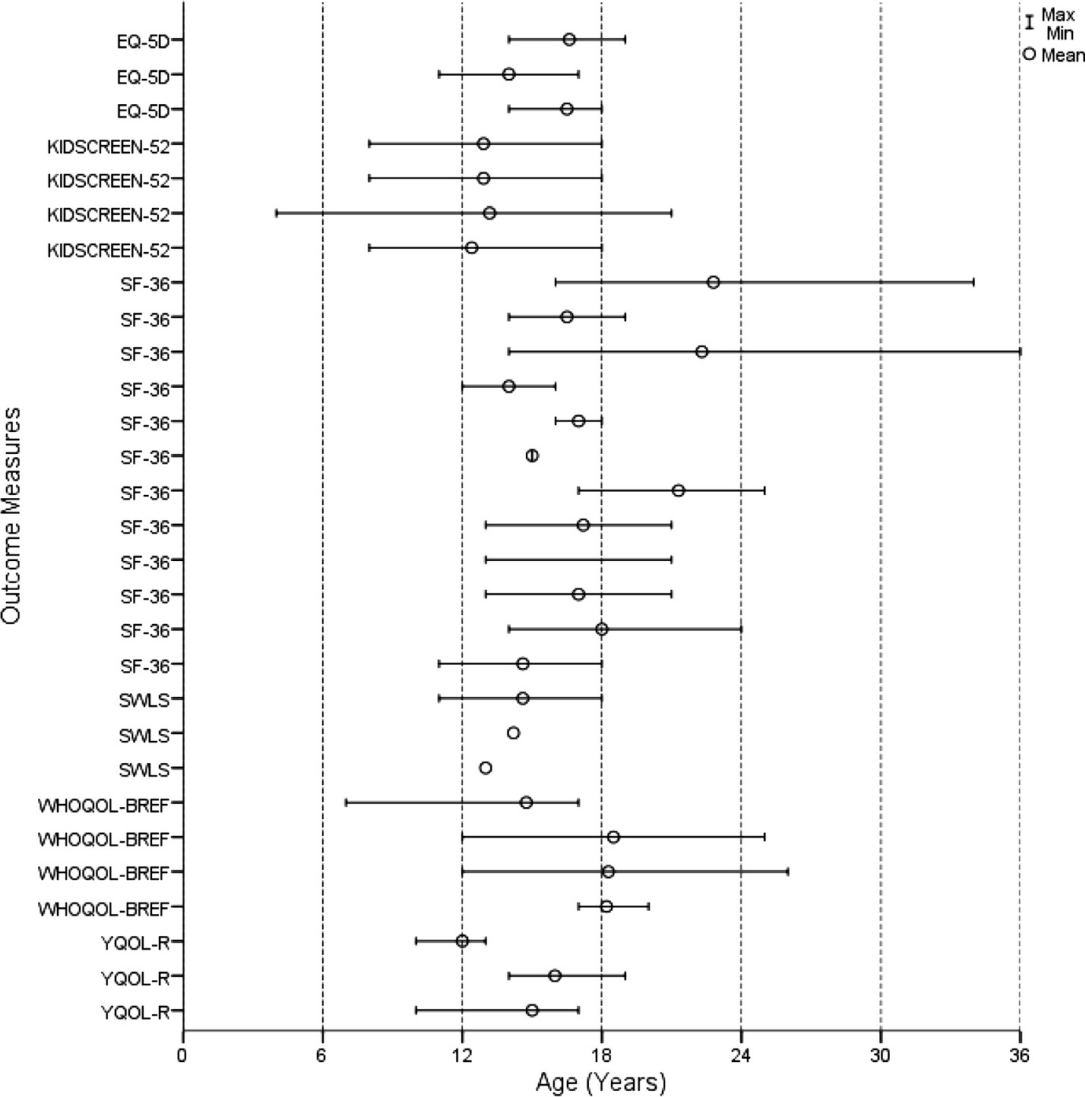
Age range and mean of quality of life measures

**Fig. 5:**
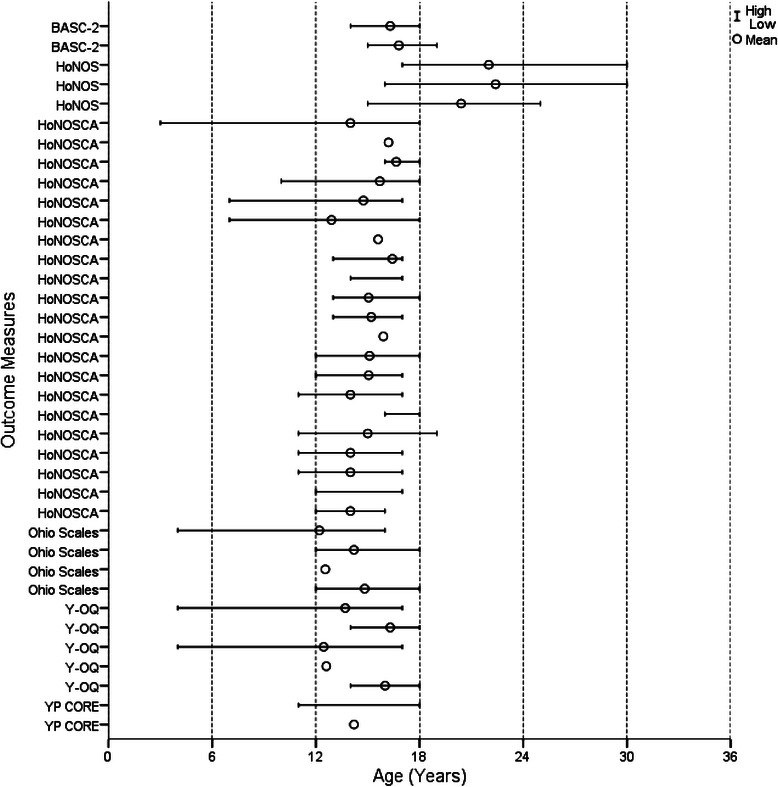
Age range and mean of multidimensional measures

Figures 2, 3, 4 and 5 show the age range and mean age for each measure in each article. Of the 29 outcome measures, 22 were used in at least one study with a sample that ranged across the age 18 child/adult demarcation point. However, only 11 of these measures were used in samples that had mean ages in both the 12 to 17 and 18 to 25 year groups. These included the BPRS, GHQ-12, K10, SCL-90-R, YSR, CGAS, CGI-S, GAF, SOFAS, SF-36, and WHOQOL-BREF. The YSR and CGAS were used predominately in the under 18 year age range. It is important to note that none of the multidimensional measures were used in samples with mean ages both above and below 18 years. There were three measures used with young people below 18 years that had an adult countermeasure used at follow-up: the YSR (YASR), CGAS (GAS), and HoNOSCA (HoNOS).

Eight measures were used across the whole 12 to 25 year age range, comprising the BPRS, GHQ-12, K10, YSR, CGI-S, GAF, SF-36 and WHOQOL-BREF. The BPRS was predominately used with samples diagnosed with psychosis and schizophrenia. The YSR was slightly modified in one article, with the term ‘kids’ changed to ‘young people’, so that it could be used across the broader 12 to 25 year age group, rather than just with those aged under 18 years. The GHQ-12 was only used in non-clinical samples, and mainly over longer time periods tracking naturalistic change. Consequently, there were only five measures deemed to be suitable for use across the whole 12 to 25 year age range and applicable to a variety of clinical and research settings and population groups: K10, CGI-S, GAF, SF-36 and WHOQOL-BREF.

### Outcome measure reporter

Outcome measures can be reported by the client (self-report), parents/carers or clinician. The review identified 13 of the 29 measures as having more than one reporter. Eight measures were self-report by the young person, which were the GHQ-12, K10, YSR, YP CORE, and all quality of life measures except the KIDSCREEN-52 and WHOQOL-BREF. Eight measures were clinician reported, comprising the HoNOS and most measures of functioning. All articles that referenced the SOFAS did not note the reporter, but this measure is clinician assessed.

Outcome measures with options for all three reporters were the SDQ, SFSS, CGI-I, and Ohio Scales. Only one of the 13 articles for the CGI-I used all three reporters, and this study aimed to explore differences between reporter types. The CBCL was the only measure with a teacher report in one article. This measure also has parent and clinician reporter versions, and a self-report version, the YSR, in the same family of measures and used in a number of the same studies. The CBCL and YSR were kept distinct, however, due to the different age ranges they target.

### Population groups

Out of the 29 outcomes measures, two were used specifically in non-clinical, community-based samples: the GHQ-12 and SWLS. The remaining 27 measures were used with various clinical participant samples, and 10 measures were used in both clinical and non-clinical samples: the CBCL, K10, SDQ, SCL-90-R, YSR, KIDSCREEN-52, SF-36, WHOQOL-BREF, BASC-2, and Y-OQ. It is important to note that all functioning measures and multidimensional measures, except the BASC-2 and Y-OQ, were used only in clinical samples.

### Intervention types

All outcome measures were used in at least one trial or treatment interventions. Many also explored naturalistic change over time in the absence of an intervention, including the CBCL, GHQ-12, K10, SCL-90-R, YSR, KIDSCREEN-52, SF-36, SWLS, and WHOQOL-BREF. The GHQ-12, KIDSCREEN-52, and SWLS were predominately used to measure naturalistic change. No measures of functioning or multidimensional mental health were used to examine naturalistic change.

### Change magnitude

The review determined whether the outcome measures were used to assess change using tests of significance, effect size, reliable change and clinical significance. All but one outcome measure (SWLS) reported change magnitude over time. There were 28 measures that reported tests of significance and 17 included effects sizes. Outcome measures showing small to medium effect sizes included the BPRS, CBCL, K10, SCL-90-R, SFSS, YSR, KIDSCREEN-52, SF-36 and YP CORE. Measures showing medium to large effect sizes included the SDQ, CAFAS, CGAS, CGI-S, GAF, EQ-5D, YQOL-R and HoNOSCA. Effect sizes of small, medium and large were based on Cohen’s d of 0.2, 0.5, and 0.8, respectively.

Of the five measures identified suitable for use with the whole 12 to 25 year range, the K10, CGI-S, GAF and SF-36 reported effect sizes. The K10 was used in one study involving 36 non-clinical participants comparing two online coping programs and a control over nine weeks. There was a significant main effect over time for all three groups, with a small effect size [48]. The CGI-S was used with 20 participants for treatment of anxiety, showing a large effect size over 14 weeks of treatment [49]. A study used the GAF with 74 psychiatric outpatient participants with a range of disorders being treated with a Mindfulness-Based Stress Reduction (MBSR) program compared to Treatment as Usual (TAU). After post treatment (8 weeks) and follow-up (3 months) a large effect size was evident for the MBSR group compared to a small decline in the TAU group [50]. Lastly, the SF-36 was used with 63 participants being treated for first-episode mania, demonstrating small effect sizes on both mental and physical component scores after 6, 12 and 18 months [51].

Only seven of the measures were analysed using a reliable change index, which included the SDQ, SCL-90-R, YSR, CGAS, GAF, Ohio scales, and Y-OQ. The SDQ, SCL-90-R, and CGAS were used in randomised trials which showed reliable change index cut-off comparisons between intervention and control groups. The SDQ, used in a randomised trial of Acceptance and Commitment Therapy (ACT) compared to TAU, showed a reliable improvement for 26 % compared to 0 % at post treatment, respectively [52]. In a randomised trial reporting reliable change using the SCL-90-R, MBSR showed 59 % of participants with no change and 41 % improved, while TAU showed 10 % worsened, 62 % had no change and 27 % improved [50]. The CGAS was used in a study of female Apache American Indians with depression, to measure outcomes for a cognitive-behaviour based program versus an education support program. Differences in reliable change between the two interventions were reported at post intervention (8 weeks), 12 weeks, 20 weeks and 32 weeks [53].

Five outcome measures used tests of both clinical significance and reliable change; namely, the SCL-90-R, YSR, GAF, Ohio Scales, and Y-OQ. The GAF was used in a young adults’ counselling centre with 78 Swedes aged 16 to 23 years with a range of mental health disorders. A reliable improvement was calculated to be an increase of at least 10 points on the GAF. At post treatment, with mean length of treatment being 11 months, 52 % of participants showed reliable improvement while 48 % showed no change. Additionally, 31 % demonstrated clinically significant improvement [54]. A study using the Y-OQ in a school-based mental health treatment program reported both reliable change and clinical significance to conclude that 45 % of clients had “recovered” by meeting both criteria [55].

### Follow-up time frame

The systematic review extracted follow-up time frames for the outcome measure studies, and categorised these as: short-term (0–6 months), medium-term (over 6 months-1 year), and long-term (over 1 year). Many measures were used across all three time frames. Measures used only in a short-term time frame included the SFSS, BASC-2, and Ohio Scales. The CGI-I, CGI-S, YQOL-R, and Y-OQ were primarily used in short-term time frames but did have some variation. Two measures primarily used in a long-term time frame were the KIDSCREEN-52 and GHQ-12. A small number of studies reported routine use of outcome measures, whereby the same measure was used at multiple time points: SDQ, SFSS, and Y-OQ.

### Sample demographics

The outcome measures were all used with a range of sample demographics, according to gender, ethnicity and socioeconomic status. All 29 outcome measures were applied in equivalent ways for males and females; only one study using the K10 identified a participant that was transgender.

The majority of studies did not report ethnicity and, for those that did, there was little consistency. There were five measures that only reported primarily Caucasian samples, but no further information on what this broad category comprised, which were the VFI, SFSS, EQ-5D, BASC-2, and Ohio Scales.

In most studies, the socioeconomic status of the sample was not reported. Outcome measures that reported being used in a lower socioeconomic sample included the CBCL, GHQ-12, SDQ, YSR, CGAS, CIS, GAF, SOFAS, VFI, and SWLS.

### Feedback systems

The review identified three outcome measures used routinely, however only two of these measures were used as part of a feedback monitoring system, the SDQ and SFSS. In each case, these measures were used repeatedly to provide routine feedback to the clinician on the young person’s outcomes. No functioning, quality of life or multidimensional measures were used as a feedback monitoring system.

The SDQ was specifically adapted in one study to be able to be used routinely in a feedback system. This session by session measure (SxS) was used to examine treatment effects using the feedback monitoring system during TAU over a year. Participants were recruited from Child and Adolescent Mental Health Services outpatient clinics, aged 11 to 19 years with a range of mental health disorders. The young clients and parents reported the SxS measure, which was fed back to clinicians and discussed with the young client. Results showed statistically significant changes after a year on the CGAS and young person reported SxS, however, no statistically significant changes in the HoNOSCA and parent reported SxS [56].

The SFSS was used in a randomised cluster controlled trial comparing weekly feedback versus no feedback monitoring system with young people being treated for a range of mental health disorders. Participants were recruited through a private health organisation, were aged 11 to 18 years and participated in the study for a mean time of 16.5 weeks. The SFSS used young person, parent and clinician reporters. Client participants with clinicians who received feedback on the SFSS improved faster than those with no feedback. Feedback effect sizes were small, being 0.18, 0.24, and 0.27 for reports from young people, clinicians, and parents, respectively. There were stronger effects when clinicians viewed multiple reporter sources; that is, from young persons, parents and clinician [12].

## Discussion

This systematic review identified 29 mental health outcome measures, reported in 184 articles examining change in mental health status for young people aged from 12 to 25 years. It is the first review to examine outcome measures specifically across this age range. Prior reviews have reflected the traditional mental health service system demarcation by focussing on outcome measures used with either children and adolescents or with adults [22, 29]. The focus of the review was on general mental health outcome measures, rather than disorder-specific symptom measures, consistent with recent research highlighting the need to measure outcomes across comorbid conditions, changing presenting problems, and different client types and settings [22, 29].

### Age range appropriate measures

There were eight outcome measures identified as being used across the whole 12 to 25 year age range. These were the BPRS, GHQ-12, K10, YSR, CGI-S, GAF, SF-36 and WHOQOL-BREF, but none are developed specifically for this target age range. Three measures are considered to be less appropriate for general youth mental health, namely: the BPRS, because it is used primarily with psychosis; the YSR, as it was specifically designed for clients under the age of 18 years; and the GHQ-12, which is mainly used with non-clinical samples to track naturalistic change. This leaves five measures deemed suitable for use across the whole 12 to 25 year age range and applicable to a variety of clinical and research settings and population groups: K10, CGI-S, GAF, SF-36 and WHOQOL-BREF. A discussion of their strengths and weaknesses follows.

The K10 was developed by Kessler and colleagues as a measure of non-specific psychological distress. It is a 10-item self-report measure which asks clients about symptoms of anxiety and depression in the past four weeks. The K10 has been widely used as a measure of mental health status in population surveys as well as an outcome measure in primary care settings. It demonstrates strong validity, excellent reliability and has been shown to be sensitive to change [29]. No studies have examined the feasibility of the K10 as a routine outcome measure; however, it has been noted as easy to use, brief and is one of the key outcome measures for the Better Outcomes in Mental Health Care Initiative in Australia [29]. In the current review, the K10 was shown to be used with clinical and non-clinical samples, tracking both treatment effects and naturalistic change. Change in the K10 was reported mainly with tests of statistical significance and a small effect size was demonstrated in one study.

The CGI-S is a brief clinician-rated global measure of current severity of the client’s symptoms and functioning. The CGI-S is one-item asking the clinician, in their clinical experience, how mentally ill the client has been over the past week from “normal” to “extremely ill” [57]. The CGI-S has been shown to be sensitive to change, showing similar change to the HoNOS. It has been identified as suitable for routine use due to its brevity and ease of administration [58]. However, there are questions about its validity and reliability and efforts have been made to improve its psychometric properties [59]. In the current review, the CGI-S was used only with clinical samples, and change was reported using statistical significance and effect size, revealing large statistical effects.

The GAF is a clinician-rated scale giving a measure of overall psychiatric disturbance integrating three dimensions of functioning: psychological, social and occupational. It is a single-item measure on a 100-point scale divided into 10-point intervals [60]. It has shown good construct and concurrent validity, but questions have been raised over its content validity. Inter-rater reliability can be low, particularly in routine clinical use [29]. It is sensitive to change when correlated with change in the Positive and Negative Syndrome Scale (PANSS) [61]. The GAF is brief, easy to use and reliability can be increased with minimal training, which makes it more acceptable in routine clinical settings [29]. In the current review, the GAF was the most frequently referenced measure, was used in only clinical samples, and showed large effect sizes and both reliable and clinically significant change. The GAF was included in the revised third and fourth editions of the Diagnostic and Statistical Manual (DSM), but removed from Version 5 in favour of the World Health Organization Disability Assessment Schedule 2.0. The DSM-5 Task Force decided that the GAF was not an adequate assessment of psychiatric functional impairment due to its lack of conceptual clarity, the need for separate assessment of severity and disability, questionable psychometrics in routine practice, and the need for specific training for proper routine clinical use [62].

The SF-36 is a multipurpose, self-report, short-form health survey containing 36 items grouped under eight scales: physical functioning, role limitation due to physical functioning, bodily pain, general health, vitality, social functioning, role limitation due to emotional problems and mental health. The eight scales can be summed into physical and mental health summary scores. The SF-36 has been used with a range of mental disorders and physical diseases, and a variety of treatments. It has been shown to be valid, reliable, sensitive to change, brief and easy to use [29]. In the current review, it was the most referenced measure of quality of life, when including its shorter 12-item version. The SF-36 was used in both clinical and non-clinical settings, over short to long-term time frames, and showed small to medium effect sizes.

The Australian WHOQOL-BREF comprises 26 items measuring broad domains of physical health, psychological health, social relationships and environment over the last two weeks. It has good validity, reliability and sensitivity to change, however, has been suggested to be more appropriate for use at a population level [29]. In the current review, it was used primarily with clinical samples over short time frames, although one study used a larger non-clinical sample tracking naturalistic change. The WHOQOL-BREF has both young people and clinician reporters, however, self-report is recommended if the client has sufficient ability to complete the measure.

These five outcome measures were used effectively in studies of samples spanning the 12 to 25 year age range, even though they were originally developed for use with adults. None of these measures has been tested specifically for its clinical utility or psychometric properties for the youth age range. The current review did not identify any outcome measures developed specifically for the adolescent and young adult demographic. While these five measures seem promising, further tests of psychometrics and clinical utility are needed.

Despite the lack of targeted measures, there were 22 out of 29 outcome measures identified in the review that were used in at least one study with a sample that ranged across the 18 years of age mental health service system demarcation point. These included measures that were originally developed to be used with young people only up to the age of 18 years, such as the CBCL, YSR, and KIDSCREEN-52 [63–65]. This reveals the need for specifically developed and targeted measures for young people. There are major developmental changes that occur for young people from the ages of 12 years, around the time of the onset of puberty, to 25 years, which is well into adulthood [66]. It is highly likely that useful measures for this age range would need some clearly defined flexibility to accommodate developmental changes, particularly in areas of psychosocial functioning such as intimate relationships, education and work.

### Type of reporter

Outcome measures can be self-report, clinician report or reported by relevant others (such as parents or teachers), and these different perspectives are all important for treatment [46]. In particular, self-report measures are essential for youth, to recognise their growing maturity and independence and engage them in their own treatment progress.

The place of parent reports may need further consideration, however, the current review identified very little use of parent reporters across the 12–25 age range, and only for children and adolescents [22]. New models of youth-focused care recognise the critical role of family, and parent reports may be relevant for clients up to 25 years of age [42], by providing another source of insight, particularly around changes in behavioural difficulties [67]. Careful attention would, however, need to be given to consent and confidentiality issues [66, 68].

### Tracking change

All the outcome measures identified in this systematic review were used to track change over time. There were eight measures used primarily within a six month period, suggesting they might be more sensitive to change in a relatively short time frame. In contrast, the KIDSCREEN-52 and GHQ-12 were used predominately in longitudinal population studies. Only three out of 29 outcome measures reported being used routinely used at multiple time points: SDQ, SFSS, and Y-OQ. Routine use of outcome measures is a necessity when used as a feedback monitoring system, and this was demonstrated in studies using the SDQ and SFSS.

Only seven outcome measures were used to report reliable change, and only five of these also reported clinically significant change. This is concerning as studies have shown that reliable and clinically significant are more clinically meaningful change measures for mental health research [13]. These methods were designed to account for measurement error and clinical thresholds, requiring change to be statistically reliable and demonstrate movement from a dysfunctional to the functional population distribution [15]. Using these criteria, individuals can be classified into the outcome categories of recovered, improved, unchanged, or deteriorated, which are meaningful and interpretable categories [16]. However, it should be noted, that calculations of reliable change and clinical significance produce more conservative change results than other approaches [16, 69]. Further, in an early intervention context, clinical significance may not be appropriate as most clients may not present in the dysfunctional range to start with. In these contexts, clinical deterioration should be monitored, however, to determine whether clients change from the functional to the dysfunctional distribution, indicating need for higher levels of intervention. More research is needed in this area to determine optimal change indices for youth outcome measures.

### Routine feedback

There has been an increase in demand for outcome measures to be used as a feedback monitoring system for clinicians [8]. Very few outcome measures were identified in the current systematic review that were used in this way, and these were designed for children and adolescents under 18 years [70]. The SDQ used young person and parent reporters and this information was fed back to clinicians to discuss with the young person. Treatment as usual with SDQ feedback showed statistically significant change on the CGAS post treatment, however, the study did not have a comparison group so it was unknown whether the change was due to the feedback, treatment as usual or the combination of both [56]. The SFSS study used young person, parent and clinician reporters and this information was fed back to clinician, but the study did not specify if this information was fed back to the young person. Feedback was found to improve client change, and this was heightened when feedback came from multiple sources [12]. Multiple feedback sources can provide different change perspectives of value to the clinician and young person client [46].

Of special note, the study using the SDQ within a feedback monitoring system showed that the measure had to be modified to be used in this way [56]. This suggests the possibility of other measures being modified or adapted to be used routinely. There are, however, several barriers to routine feedback, which may account for the small number of measures identified here [2, 71]. These include constraints around time, resources and training needed, and perceived lack of clinical utility [72]. There are likely to be additional barriers for young people as clients, as they are a unique client group with higher dropout rates, are often referred by parents or teachers rather than being self-referred, and have different goals for therapy and therapeutic expectations compared with adults [20].

### Limitations

A thorough search strategy was employed in this systematic review and it identified a large number of outcome measures and studies, but it is possible that relevant measures were missed. Notably, article authors were not contacted for additional information and the methodology excluded articles that were not written in English, meaning measures used specifically in other cultures were excluded. The eligibility criteria also excluded articles pertaining to participants with other health conditions, including substance use and situational stressors. This was done partly to make the review more manageable, but may have excluded relevant measures. Only two databases were used in the search strategy, MEDLINE and PsychINFO, although these are the most commonly used in systematic reviews of mental health outcome measures [22, 45, 73]. Together, the databased yielded an initial 11920 articles, which was filtered to a comprehensive 184 studies, identifying 29 outcome measures. Nevertheless, some measures, especially those not often used for research purposes and primarily used in clinical practice, may have been missed.

In particular, some popular outcome measures were not identified via the final criteria, including the Depression Anxiety Stress Scale (DASS) and the Outcome Rating Scale (ORS). The DASS is a self-report measure which comes in a 21 or 42-item version [74]. It is commonly used as individual scores for depression, anxiety and stress and, therefore, was excluded as measuring specific mental health conditions. The ORS is an outcome measure developed as a brief alternative to the Outcome Questionnaire 45.2 (OQ-45.2) [75]. The Y-OQ, which was included in this review, also comes from the same family of measures. There is a growing body of research around the ORS, particularly regarding its use as a feedback monitoring system for clinicians [76]. However, in this review, it was excluded as it was unique to only one study with young people aged 12 to 25 years [77].

## Conclusions

Mental health outcome measures are essential for quality assurance and monitoring the effectiveness of services, and for tracking longitudinal health trends across time [5, 6]. Although this review identified a large number of measures used with young people aged 12 to 25 years, only eight were used across this whole age range, each with strengths and weaknesses. Overall, the review found no measures designed specifically for young people. There is a growing push for outcome measures to be routinely used as feedback monitoring systems, and to determine clinically meaningful change [7, 20]. Only two measures were identified here as being used in this way and this is an area of particular research need for youth mental health because of the potential for such an approach to benefit clients [12]. Future research should focus on development of mental health outcome measures designed specifically for young people aged 12 to 25 years to accompany changes in mental health services that target this age range. The measures should be sensitive to reliable and possibly clinically significant change that is meaningful to young people, and also suitable for routine use as feedback to clinicians and young people themselves. This will provide services with age-appropriate measures with better clinical utility and comparative usefulness to drive delivery of the better mental health outcomes for young people, who have such a heightened need for early and effective mental health care.

## Additional files

Additional file 1: **PRISMA checklist.** (PDF 192 kb)
Additional file 2: **Mental health outcome measures used with young people 12 to 25 years [**78**–**248**].** (XLSX 46 kb)

## Abbreviations

ACT: Acceptance and Commitment Therapy
ASR: Adult Self-Report
BASC-2: Behavioural Assessment System for Children-2
BPRS: Brief Psychiatric Rating Scale
CAFAS: Child and Adolescent Functional Assessment Scale
CBCL: Child Behaviour Check List
CGAS: Children’s Global Assessment Scale
CGI-I: Clinical Global Impressions Scales-Improvement scales
CGI-S: Clinical Global Impressions Scales-Severity of Illness
CIS: Columbia Impairment Scale
DASS: Depression Anxiety Stress Scale
DSM: Diagnostic and Statistical Manual
EQ-5D: EuroQol
GAF: Global Assessment of Functioning
GAS: Global Assessment Scale
GHQ-12: General Health Questionnaire-12
HoNOS: Health of the Nation Outcome Scale
HoNOSCA: Health of the Nation Outcome Scales for Children and Adolescents
K10: Kessler Psychological Distress Scale
MBSR: Mindfulness-Based Stress Reduction
OQ-45.2: Outcome Questionnaire 45.2
ORS: Outcome Rating Scale
PANSS: Positive and Negative Syndrome Scale
PRISMA: Preferred Reporting Items for Systematic Reviews and Meta-Analyses
SCL-90-R: Symptom Checklist 90 Revised
SDQ: Strengths and Difficulties Questionnaire
SF-12: Medical Outcomes Study (MOS) 12-item Short Form Health Survey
SF-36: Medical Outcomes Study (MOS) 36-item Short Form Health Survey
SFSS: Symptom and Functioning Severity Scale
SOFAS: Social and Occupational Functioning Assessment Scale
SWLS: Satisfaction with Life Scale
SxS: Session by session measure
TAU: Treatment as Usual
VFI,: Vanderbilt Functioning Index
WHOQOL-BREF: World Health Organisation Quality of Life Instrument-Brief
Y-OQ: Youth Outcome Questionnaire
YASR: Young Adult Self-Report
YOQ-30: Youth Outcome Questionnaire-30
YP CORE: Young Persons Clinical Outcomes for Routine Evaluation questionnaire
YQOL-R: Youth Quality of Life Instrument-Research Version
YSR: Youth Self-Report
